# Sertraline as an Antidepressant and Inflammatory Cytokines in Depressive Disorders: A Systematic Review and Meta-Analysis of Randomized Double-Blind Placebo-Controlled Trials

**DOI:** 10.3390/medsci14040460

**Published:** 2026-08-06

**Authors:** Rafaela F. Rossetto, Julia S. Pissocaro, Davi A. Cavalheri, Rodrigo D. Raimundo, Sandra Maria Barbalho, Andrey A. Porto, David M. Garner, Vitor E. Valenti

**Affiliations:** 1 Systematic Reviews Center for Cardiovascular and Metabolic Health, School of Philosophy and Sciences, São Paulo State University (UNESP), Av. HyginoMuzzi Filho, 737, Marília 17525-900, SP, Brazil; rafaela.rossetto@unesp.br (R.F.R.); julia.scariot@unesp.br (J.S.P.); davi.cavalheri@unesp.br (D.A.C.); 2 Laboratório de Delineamento de Estudos e Escrita Científica, Centro Universitário, Faculdade de Medicina do ABC (FMABC), Santo André 09060-870, SP, Brazil; rodrigo.raimundo@fmabc.br; 3 Department of Biochemistry and Pharmacology, School of Medicine, Universidade de Marília (UNIMAR), Marília 17525-902, SP, Brazil; sandra.barbalho@fatec.sp.gov.br; 4 Postgraduate Program in Structural and Functional Interactions in Rehabilitation, School of Medicine, Universidade de Marília (UNIMAR), Marília 17525-902, SP, Brazil; 5 Department of Biochemistry and Nutrition, School of Food and Technology of Marília (FATEC), Marília 17500-000, SP, Brazil; 6 Department of Pharmacology, School of Medicine of Ribeirao Preto, Faculdade de Medicina de Ribeirão Preto/Universidade de São Paulo—FMRP/USP, Ribeirao Preto 14049-900, SP, Brazil; aa.porto@unesp.br; 7 Cardiorespiratory Research Group, School of Biological and Medical Sciences, Faculty of Health and Life Sciences, Oxford Brookes University, Headington Campus, Gipsy Lane, Oxford OX3 0BP, UK; davidmgarner1@gmail.com

**Keywords:** sertraline, cytokines, inflammation, depression, meta-analysis

## Abstract

**Background:** Sertraline, a selective serotonin reuptake inhibitor widely prescribed for depressive disorders, has been proposed to exert immunomodulatory effects through modulation of inflammatory pathways; however, clinical evidence regarding its effects on circulating cytokines remains inconsistent. This systematic review and meta-analysis evaluated the effects of sertraline on inflammatory cytokines in adults. **Methods:** Searches were conducted in LILACS, CINAHL, MEDLINE/PubMed, Cochrane Library, Scopus, and Web of Science to identify randomized single- or double-blind placebo-controlled trials assessing sertraline and inflammatory biomarkers. Ten studies met the eligibility criteria. Random-effects meta-analyses were performed for interleukin-6 (IL-6), interleukin-10 (IL-10), and tumor necrosis factor-α (TNF-α). **Results:** Sertraline did not significantly reduce circulating IL-6 (MD −1.06, 95% CI −2.32 to 0.19; I^2^ = 86%; *p* = 0.10), IL-10 (MD < 0.01, 95% CI −0.23 to 0.23; I^2^ = 72%; *p* = 0.98), or TNF-α levels (MD −0.84, 95% CI −4.04 to 2.37; I^2^ = 72%; *p* = 0.61). Heterogeneity was substantial, all studies showed high overall risk of bias, and the certainty of evidence was very low for all outcomes. **Conclusions:** Current evidence does not support a significant or reliable anti-inflammatory effect of sertraline on circulating cytokines, and further well-designed trials using standardized inflammatory assessments are warranted.

## 1. Introduction

Major depressive disorder (MDD) is one of the most widespread and disabling psychiatric disorders globally and represents a major public health challenge owing to its substantial emotional, social, and economic burden. Depression affects hundreds of millions of individuals worldwide internationally and is related with impaired quality of life, reduced occupational and social functioning, increased healthcare utilization, and elevated mortality rates, including suicide risk [1]. Besides its psychiatric manifestations, depression has also been linked to several chronic medical conditions, including cardiovascular disease, diabetes mellitus, obesity, autoimmune disorders and chronic kidney disease (CKD). The coexistence of depression with these conditions suggests that common biological mechanisms may contribute to both psychiatric and systemic manifestations [2].

Even though the pathophysiology of depression is multifactorial and not yet fully explained, accumulating evidence over recent decades has increasingly implicated systemic inflammation and immune dysregulation as significant contributors to the development and progression of depressive disorders. Several clinical studies have consistently reported elevated circulating levels of pro-inflammatory cytokines and inflammatory biomarkers in individuals with depression, including IL-6, tumor necrosis factor-α (TNF-α), interleukin-1 beta (IL-1β), and C-reactive protein (CRP) [3,4,5,6,7]. Moreover, increased inflammatory activity has been linked with greater symptom severity, treatment resistance, cognitive impairment, fatigue, anhedonia and poorer clinical outcomes [8].

The connection between depression and inflammation appears to be bidirectional. chronic psychological stress and depressive states may activate inflammatory pathways through dysregulation of the hypothalamic–pituitary–adrenal (HPA) axis and autonomic nervous system [9,10]. Inflammatory mediators may also directly influence brain function and behavior. Pro-inflammatory cytokines can cross the blood–brain barrier or communicate with the central nervous system through neural and humoral pathways, thereby affecting neurotransmitter systems involved in mood regulation. This is clinically relevant because increased serotonergic neurotransmission is one of the principal mechanisms through which selective serotonin reuptake inhibitors, including sertraline, alleviate depressive symptoms. By increasing synaptic serotonin availability, these drugs may help restore signaling within neural circuits related to mood, emotional processing, reward, and stress regulation, which are commonly disrupted in depressive disorders, thus changing neurotransmitter metabolism, neuroendocrine function, and synaptic plasticity [11]. Cytokines may reduce serotonin bioavailability by activating indoleamine 2,3-dioxygenase and the kynurenine pathway, thereby diverting tryptophan metabolism away from serotonin synthesis and toward neuroactive kynurenine metabolites, including 3-hydroxykynurenine and quinolinic acid, which have been described as neurotoxic metabolites [12].

Furthermore, inflammatory processes may damage dopaminergic signaling, increase oxidative stress, disrupt mitochondrial function, and lessen levels of neurotrophic factors for instance brain-derived neurotrophic factor (BDNF), ultimately encouraging neurodegeneration and impaired neuroplasticity [13]. These mechanisms may help elucidate the overlap between inflammatory activation and several core depressive symptoms, with low mood, fatigue, cognitive dysfunction, sleep disturbances, and loss of motivation [1]. From a clinical perspective, sertraline is often regarded as one of the more favorable selective serotonin reuptake inhibitors (SSRIs) for patients with relevant somatic comorbidities. Randomized evidence supports its cardiovascular safety in patients with recent myocardial infarction or unstable angina, and its use has also been evaluated in individuals with CKD not requiring dialysis [14,15]. Sertraline is also commonly considered during pregnancy and breastfeeding when pharmacological treatment is clinically indicated because of its comparatively well-established reproductive safety profile and low transfer into breast milk. Nevertheless, treatment decisions during pregnancy and lactation require an individualized assessment of maternal benefits and potential fetal or neonatal risks. Consequently, increasing attention has been focused towards understanding whether anti-inflammatory mechanisms may in part mediate the therapeutic effects of antidepressant treatments.

Amongst the currently available antidepressants, SSRIs remain the first-line pharmacotherapy for MDD because of their favorable efficacy and safety profile. Sertraline, especially, is one of the most widely prescribed SSRIs worldwide and is usually prescribed for the treatment of depression, anxiety disorders, obsessive–compulsive disorder, post-traumatic stress disorder, and other psychiatric conditions. Sertraline principally acts by inhibiting serotonin reuptake at the synaptic cleft, thus increasing serotonergic neurotransmission and improving depressive symptoms [16]. Yet, beyond its central serotonergic effects, increasing evidence suggests that sertraline may also affect immune and inflammatory pathways. Experimental studies have suggested several mechanisms through which sertraline may exert immunomodulatory effects. Sertraline may reduce the production of proinflammatory cytokines, attenuate microglial activation, modulate T-cell responses, and reduce oxidative stress [17,18,19,20,21]. Some research has similarly suggested that SSRIs may normalize HPA axis dysfunction and improve neuroplasticity through interactions with inflammatory pathways [22]. Furthermore, reductions in inflammatory biomarkers after sertraline treatment have been reported in some clinical populations, mainly amongst patients with elevated baseline inflammation or comorbid cardiovascular and metabolic disorders [23]. These findings have led to the hypothesis that anti-inflammatory mechanisms may contribute, at least in part, to the therapeutic effects of sertraline and other antidepressants.

Despite increasing scientific interest in this area, the available evidence about the effects of sertraline on inflammatory biomarkers remains highly heterogeneous and questionable. Some randomized controlled trials have established significant reductions in inflammatory markers for example IL-6, TNF-α, and CRP after sertraline treatment [23,24], but other studies have failed to observe significant immunological changes despite enhancements in depressive symptoms [25,26]. Still, it was advised that baseline inflammatory status may predict antidepressant response, where others found no connection between inflammatory biomarkers and clinical outcomes [27]. In this sense, the interpretation of the current research literature is further complicated by important methodological and clinical heterogeneity among studies, including differences in participant characteristics, treatment duration, sertraline dosage, concomitant therapies, laboratory methods, and inflammatory outcome assessment.

Another important limitation of the existing scientific research literature is the lack of comprehensive evidence synthesis using rigorous methodological standards. Even if individual studies have explored the affiliation between sertraline and inflammatory biomarkers [24,25], no previous review has comprehensively synthesized evidence solely from randomized double-blind placebo-controlled trials while quantitatively evaluating the effects of sertraline on circulating cytokines. A systematic review and meta-analysis may thus provide a more robust and reliable assessment of the available evidence, permitting identification of consistent trends, evaluation of heterogeneity, and assessment of methodological quality and certainty of evidence. Such an approach is particularly important given the growing clinical interest in inflammation as a therapeutic target in psychiatric disorders and the potential implications for personalized treatment strategies.

Hence, the present study aimed to systematically review and quantitatively synthesize evidence from randomized, double-blind, placebo-controlled trials evaluating the effects of sertraline on circulating inflammatory cytokines in adults. Explicitly, this systematic review and meta-analysis studied the effects of sertraline on key inflammatory biomarkers, including IL-6, IL-10, and TNF-α, while also assessing methodological quality, risk of bias, heterogeneity, and certainty of evidence across the included studies.

## 2. Materials and Methods

### 2.1. Protocol and Registration

This review adhered to the guidelines of the Preferred Reporting Items for Systematic Reviews and Meta-Analyzes (PRISMA) [28] (see Table S1). It is registered in the PROSPERO database under the registration number CRD420251170714.

### 2.2. Eligibility Criteria

We included studies from peer-reviewed scientific journals published from the inception of the database up to July 2026. The eligibility criteria were defined according to the PICOS framework: Population, Intervention, Comparator, Outcomes, and Study Design, explicitly including:

(P) Adults (≥18 years) who were prescribed for sertraline treatment. Exclusion criteria: Animal or in vitro studies, participants under 18 years old;

(I) Treatment with sertraline (any dose or duration) as monotherapy or combined therapy. Exclusion criteria: Non-placebo comparators which did not allow the evaluation of the effects of sertraline;

(C) For comparison groups, we included studies that assessed subjects that received placebo;

(O) Primary: Inflammatory cytokine markers (e.g., IL-6, TNF-α, IL-1β, IL-10, or other cytokines/biomarkers of systemic inflammation). Secondary: Clinical symptom changes (e.g., mood symptoms, relapse rate), if stated alongside inflammatory markers;

(S) We included studies with single or double-blind randomized controlled trials and crossover designs. This review is limited to articles published in peer-reviewed scientific journals, master’s theses and doctoral dissertations. We omitted conference abstracts, descriptive studies, case reports, editorials, and reviews.

### 2.3. Information Source, Search Strategy and Study Selection

Searches were performed across Lilacs, CINAHL, MEDLINE/PubMed (National Library of Medicine), Cochrane, Scopus, and Web of Science databases. The search terms were: “Sertraline” OR “Zoloft” OR “Lustral” OR “Altruline” OR “Gladem” OR “Sealdin” AND “Cytokine” OR “Interleukin” (see Table S2: Search strategy for details).

All retrieved records were imported into Rayyan QCRI for duplicate removal and study screening (Qatar Computing Research Institute, Doha, Qatar) for duplicate exclusion. The initial screening involved reading the titles and abstracts within Rayyan. Next, the full articles were judged for appropriateness by at least two, and up to four, independent reviewers. A third reviewer was consulted to resolve any disagreements regarding inclusion. Following study selection, the authors assessed whether the available data were sufficiently comparable for quantitative synthesis.

### 2.4. Data Collection and Data Extraction

Data extraction was executed independently by at least two reviewers. Information about the author, study design, participant characteristics, and intervention details was extracted from the primary studies and compiled in a Table. Missing data were sought by contacting the corresponding study authors. In cases where the authors did not respond, the Web Plot Digitizer^®^, version 5 was used to extract data presented graphically. Inflammatory data were logged as mean and standard deviations (SD). Values initially presented as “standard error” or “confidence intervals” (CI) in the primary studies were converted to SD.

### 2.5. Data Items

Inflammatory marker data were logged to compare outcomes before and after the intervention. Information on the participant and intervention profiles, in addition to funding sources, was extracted from the selected references. Any absent or unclear information was omitted [28].

### 2.6. Assessment of the Risk of Bias in Individual Studies and Across Studies

The risk of bias analysis was completed using the Cochrane Risk of Bias tool [29] (Sterne et al., 2019) and achieved within the Review Manager program (RevMan 5.4.1). This domain-based tool gauges bias across six key areas: “Randomization process,” “Deviations from intended interventions,” “Missing outcome data,” “Measuring of the outcome,” “Selection of the reported results,” and “Overall bias.”

The assessment for each domain was regarded as “low risk,” “some concerns,” or “high risk,” based on the specific judgment criteria outlined in the table developed by Sterne et al. [29] (“Reviewers’ judgment and criteria for judgment”).

Two independent authors performed the risk of bias analysis, and a third researcher was consulted to resolve any inconsistencies in their judgments. The assessors received suitable educational training sessions before conducting the analysis.

We also identified other variables that could impact the cumulative evidence, such as publication bias or selective reporting within studies.

### 2.7. Certainty Assessment

The certainty of the evidence was evaluated by means of the Grades of Recommendation, Assessment, Development, and Evaluation (GRADE) Working Group approach [30] (GRADE Working Group, 2004). This analysis considered the study design of randomized trials (representing strong evidence). Also, the strength of the evidence analysis incorporated considerations of study quality (including detailed study methods and execution) and any limitations [30]. The summary of the findings table was created using GRADEpro GDT v4^®^ (McMaster University, Hamilton, ON, Canada).

### 2.8. Qualitative Analysis

A narrative synthesis was performed to feature the methodology and outcomes of each included study. Individual study results, with the results of the qualitative analysis of inflammatory parameters for both intervention and control protocols, are offered in the text and tables.

### 2.9. Synthesis of Results and Summary Measures

Following the selection of all references, we judged the feasibility of carrying out a meta-analysis. If fitting, the outcome values were extracted. For the meta-analysis construction, data from the post-intervention period were mandatory. We adhered to the criterion of extracting all available between-group data in the post-intervention phase.

Heterogeneity was measured via the I^2^ statistic. The following thresholds were necessary for interpretation:

- 0–29% indicated no important heterogeneity;
- 30–49%, moderate heterogeneity;
- 50–74%, substantial heterogeneity; and
- 75–100%, considerable heterogeneity [31,32].

For the 95% CI and the Test for overall effect size, a significant difference was assumed if *p* < 0.05 (or <5%). If studies required dispersion values for change (such as SD, 95% CI, Standard Errors, or *p*-values), the absent SDs of the changes (SD) were calculated. Meta-analysis outcomes were presented as weighted Mean Difference (MD), 95% CI, and *p*-value. An overall MD for the intervention group compared to the control group was considered statistically significant if *p* < 0.05. The results were exhibited in forest plots. [33]. All data manipulations were performed using the Review Manager Program (RevMan 5.4.1).

## 3. Results

### 3.1. Study Selection

A total of 935 records were recognized through database searches. After the exclusion of 195 duplicate records, 740 unique records were screened as said by the predefined inclusion criteria. Following title and abstract screening, 695 records were omitted.

Next, 45 reports were sought for retrieval. Of these, 42 full-text reports were positively assessed for eligibility, while three could not be fully evaluated, including two conference abstracts and one report that was not retrieved. After full-text assessment, 32 reports were omitted for the subsequent reasons: sertraline combined with other therapy (n = 11), non-randomized controlled trial design (n = 7), absence of a control/placebo group (n = 11), letter (n = 1), and no outcome of interest (n = 2).

**Figure 1:**
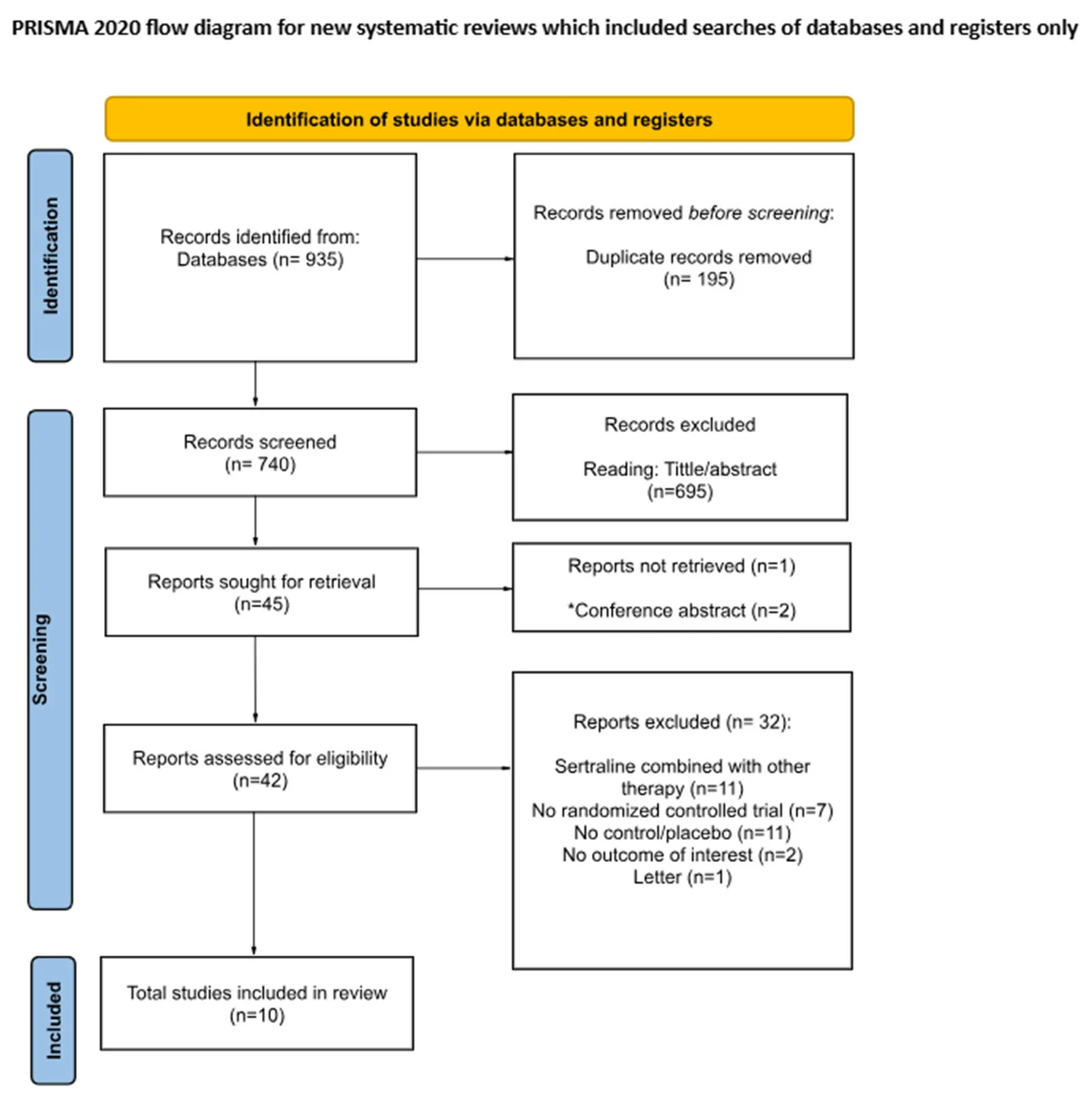
PRISMA 2020 flow diagram of the study selection process. * References published in Conference as Abstracts.

Accordingly, 10 studies were included in the final review. The search strategy and study selection process were conducted according to the PRISMA 2020 statement, as illustrated in Figure 1. Detailed information on the studies included is offered in Table 1.

### 3.2. Results of Individual Studies

Ardakani et al. [24] randomized 44 participants to sertraline (n = 24) or placebo (n = 20). Sertraline was administered orally once daily for 8 weeks at a fixed dose, although the exact dosage was not reported. The control group received an oral placebo according to the same schedule under double-blind conditions. Serum IL-6 and IL-10 were evaluated. Sertraline significantly reduced IL-6 levels from baseline and compared with placebo, with a significantly greater change at week 8 (*p* = 0.048). In contrast, IL-10 did not differ significantly within or between groups. These findings suggest a possible effect of sertraline on IL-6, although the absence of a reported dose limits the methodological characterization of the intervention.

Blumenthal et al. [34] included patients with coronary heart disease and allocated them to aerobic exercise (n = 37), sertraline (n = 40), or placebo (n = 24). Sertraline was initiated at 50 mg/day and titrated to 50–200 mg/day according to clinical response and tolerability; the median dose was 50 mg/day. Treatment was administered orally once daily for 16 weeks. The placebo tablets were identical in appearance and followed the same titration and monitoring schedule, with allocation concealed from participants, investigators, and the treating psychiatrist; only the research pharmacist was aware of treatment assignment. Both aerobic exercise and sertraline reduced depressive symptoms compared with placebo, and exercise improved aerobic capacity and exercise tolerance. However, sertraline did not significantly alter IL-6 or other inflammatory markers relative to placebo. No significant changes were observed in body weight, body mass index, endothelial function, platelet activity, or most other cardiovascular measures, although active treatment groups showed a nonsignificant trend toward improved heart rate variability.

Bot et al. [26] analyzed 122 adults with documented coronary heart disease and current major depressive disorder diagnosed according to the Diagnostic and Statistical Manual of Mental Disorders, Fourth Edition criteria. The sample comprised 41 women and 81 men. All participants received sertraline at a fixed dose of 50 mg/day for 10 weeks, with adherence exceeding 98%. Participants were additionally assigned to omega-3 or matching placebo capsules, which were identical in appearance, and participants, investigators, and research staff were blinded to this allocation. CRP, IL-6, and TNF-α were examined as potential predictors of antidepressant response. Baseline levels did not differ between responders and nonresponders or between remitters and nonremitters and did not consistently predict treatment outcomes. Although depressive symptoms improved, CRP and IL-6 increased over time, whereas TNF-α remained unchanged. Because all participants received sertraline, this study primarily evaluated inflammatory biomarkers as predictors of response rather than the direct effect of sertraline versus placebo.

Brunoni et al. [35] initially randomized 120 antidepressant-free adults with unipolar major depressive disorder who were experiencing an acute nonpsychotic depressive episode. Cytokine analyses were restricted to 73 participants with complete baseline and endpoint samples. Participants received sertraline at a fixed dose of 50 mg/day, matching placebo, transcranial direct current stimulation, sham stimulation, or combinations of these interventions for 6 weeks. Sertraline and placebo were administered orally once daily, and both participants and investigators were blinded to pill assignment. The assessed cytokines were IL-2, IL-4, IL-6, IL-10, IL-17A, interferon-γ, and TNF-α. Plasma concentrations of all cytokines except TNF-α decreased over time; however, these reductions were similar across the sertraline, placebo, transcranial direct current stimulation, and combined-treatment groups and were not associated with clinical response. Thus, the observed cytokine changes were not specific to sertraline and may have reflected time-related, nonspecific, or placebo effects.

Using the same original randomized sample, Brunoni et al. [36] evaluated soluble TNF receptors in 73 antidepressant-free adults with moderate-to-severe, nonpsychotic unipolar major depressive disorder and complete blood samples. Sertraline was administered orally at 50 mg/day for 6 weeks, either alone or concurrently with transcranial direct current stimulation, and was compared with a matching placebo pill under blinded conditions. Soluble TNF receptor 1 and soluble TNF receptor 2 levels did not change significantly over time and did not differ among sertraline, placebo, transcranial direct current stimulation, or combined-treatment groups. Baseline receptor concentrations also did not predict antidepressant response. This study differed from the other trials by focusing on soluble TNF receptors rather than circulating cytokines themselves and by evaluating a factorial pharmacological and neuromodulatory intervention.

Gregg et al. [25] randomized 193 adults with stage 3–5 CKD not requiring dialysis and comorbid major depressive disorder to sertraline (n = 97) or placebo (n = 96). Participants had moderate-to-severe depressive symptoms and a substantial burden of systemic inflammation related to CKD. Sertraline was administered orally once daily for 12 weeks according to the CAST protocol, beginning at 50 mg/day and increasing to a maximum of 200 mg/day based on tolerability and clinical response. The matching placebo tablets were identical in appearance and were administered under the same double-blind schedule and clinical monitoring procedures. High-sensitivity IL-6, albumin, and prealbumin were evaluated. IL-6 did not independently predict response to sertraline, and treatment did not significantly reduce inflammatory biomarker levels over time. No significant differences were observed between groups in improvement, response, or remission rates. In this trial, inflammatory measures were primarily examined as predictors of treatment response rather than solely as treatment-mediated outcomes.

Khameslo et al. [37] included 33 male patients who had undergone first-time coronary artery bypass graft surgery. Participants were allocated to placebo (n = 8), sertraline (n = 8), aerobic exercise plus placebo (n = 8), or aerobic exercise plus sertraline (n = 9). Sertraline was administered orally at a fixed dose of 50 mg/day for 8 weeks alongside usual cardiac medications. The control tablets contained 50 mg/day placebo, were identical in appearance to sertraline, and were administered once daily under double-blind conditions. IL-6 and TNF-α were the principal proinflammatory biomarkers assessed. Sertraline alone significantly reduced both markers compared with placebo, whereas the combination of aerobic exercise and sertraline generally produced the greatest improvements. Neurotrophic factors, including brain-derived neurotrophic factor and nerve growth factor, also increased, and depression and anxiety scores decreased in the active intervention groups. This small factorial trial was methodologically distinct because it evaluated the independent and combined effects of sertraline and exercise in a postsurgical cardiac population.

Pizzi et al. [38] randomized 100 adults with documented coronary heart disease and clinically significant depressive symptoms, defined by a Beck Depression Inventory score of at least 10. Ninety-five participants completed the study, including 47 in the sertraline group and 48 in the placebo group. Sertraline was administered orally at 50 mg/day for the first 6 weeks and could be titrated to a maximum of 200 mg/day between weeks 7 and 12 according to response and tolerability; the final mean dose was 70 ± 39 mg/day. Treatment continued for 20 weeks, and adherence was monitored by pill counts. The placebo tablets were identical in appearance and followed the same dosing schedule under double-blind conditions. Sertraline significantly reduced depressive symptoms and IL-6 levels compared with baseline and placebo. Reductions in CRP and fibrinogen and improvements in flow-mediated dilation were also reported, whereas endothelium-independent vasodilation remained unchanged. This was the longest included intervention and incorporated inflammatory, vascular, and depressive outcomes.

Taraz et al. [23] assessed 101 adults for eligibility and randomized 50 patients with end-stage renal disease receiving maintenance hemodialysis for at least 3 months and depression identified using the Beck Depression Inventory-II. Twenty-five participants were allocated to sertraline and 25 to placebo; 21 and 22 participants, respectively, completed the 12-week intervention. Sertraline was administered orally at 50 mg/day for the first 2 weeks and increased to 100 mg/day for the remaining 10 weeks. Adherence was monitored by pill counts, and matching placebo tablets were administered according to the same once-daily double-blind schedule. IL-6, TNF-α, and IL-10 were evaluated. IL-6 decreased significantly in the sertraline group compared with placebo at week 12 (*p* < 0.01). TNF-α decreased within the sertraline group but did not differ significantly between groups, whereas IL-10 increased within the sertraline group without a significant between-group difference. Serum albumin and other biochemical measures remained unchanged. This study was distinguished by its hemodialysis population and the use of a renal-safety-oriented dosing regimen.

Tucker et al. [39] screened 107 individuals and randomized 59 adults with chronic posttraumatic stress disorder (PTSD) diagnosed according to Diagnostic and Statistical Manual of Mental Disorders, Fourth Edition criteria and a Clinician-Administered PTSD Scale score of at least 50. The intent-to-treat sample included 58 participants: 18 received sertraline, 19 received citalopram, and 7 received placebo; an additional group of 21 trauma-exposed mentally healthy individuals served as a nonmedicated comparison group. Sertraline was administered orally once daily at a flexible dose of 50–200 mg/day for 10 weeks, with a mean final dose of 134.1 mg/day. The dose was titrated according to clinical response and tolerability. Inert placebo tablets were identical in appearance to the active selective serotonin reuptake inhibitors, and treatment allocation was concealed from participants and investigators. IL-1β and soluble IL-2 receptor were assessed. PTSD and depressive symptoms decreased across the sertraline, citalopram, and placebo groups. IL-1β decreased and soluble IL-2 receptor increased across treatment groups, reaching levels comparable to those of healthy controls. Cortisol remained largely unchanged, except for an increase in afternoon cortisol in the placebo group. This study differed from the others by including an active antidepressant comparator, a healthy trauma-exposed group, and a population with PTSD rather than major depressive disorder.

### 3.3. Synthesis of Results

**Figure 2:**
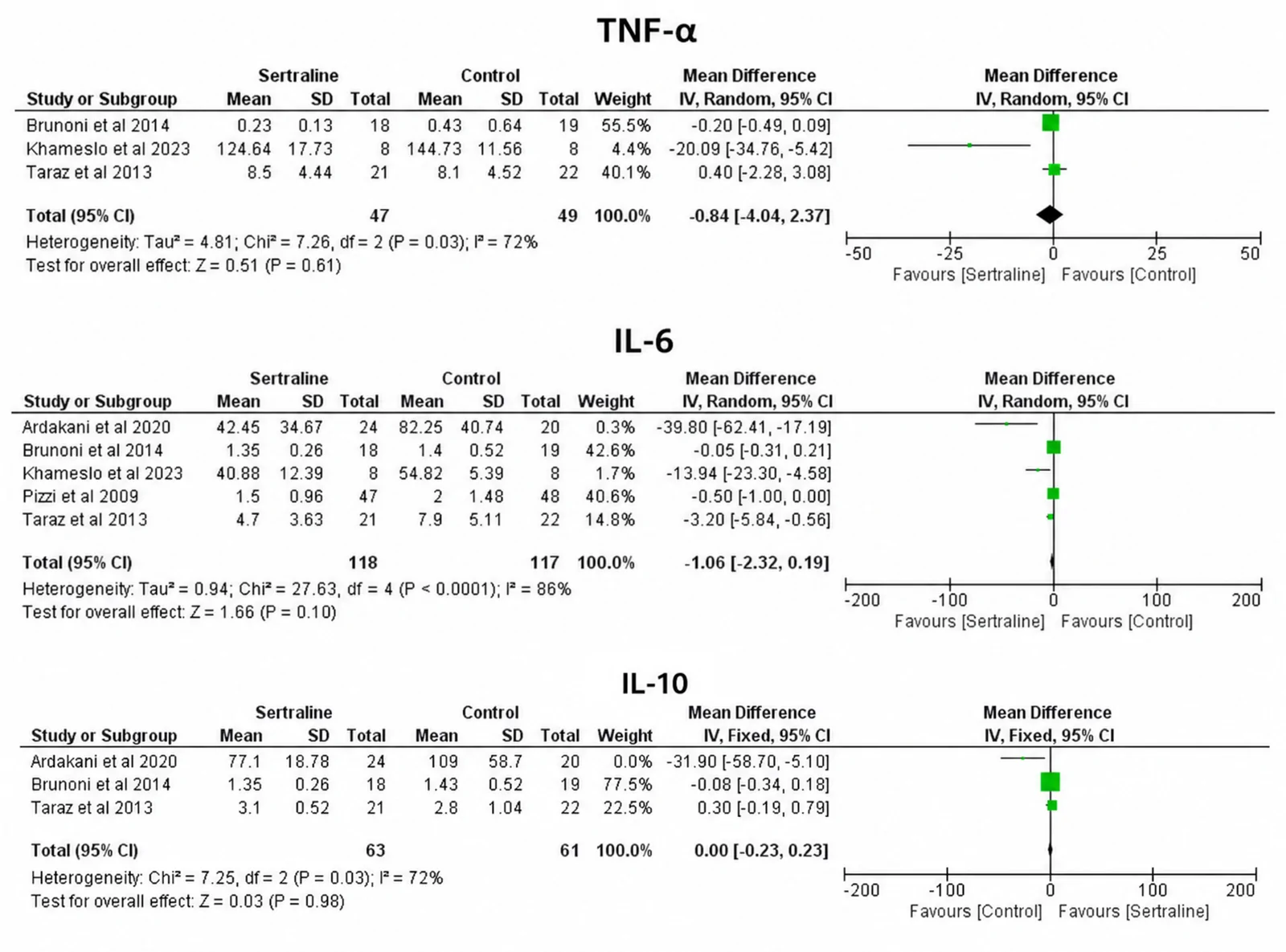
Forest plots of the effects of sertraline on IL-6, IL-10, and TNF-*α*. Taraz et al. 2013 [23], Ardakani et al. 2020 [24], Brunoni et al. 2014 [35], Khameslo et al. 2023 [37] and Pizzi et al. 2009 [38].

The meta-analysis assessed the effects of sertraline on inflammatory biomarkers, including IL-6, IL-10, and TNF-α, across the included studies (Figure 2).

For IL-6, the meta-analysis included 235 participants from five studies. The pooled analysis showed no statistically significant overall effect of sertraline, with a mean difference (MD) of −1.06 (95% CI: −2.32, 0.19; Z = 1.66, *p* = 0.10). Still, substantial heterogeneity was observed (I^2^ = 86%), indicating considerable variability among studies, which may reflect changes in patient populations, clinical conditions, treatment duration, or methodological approaches (Figure 2).

For IL-10, three studies comprising 124 participants were included in the analysis. The pooled effect indicated no significant difference between the sertraline and control groups, with an MD of <0.01 (95% CI: −0.23, 0.23; Z = 0.03, *p* = 0.98). Heterogeneity was high (I^2^ = 72%), signifying notable inconsistency across the included studies despite the overall null effect (Figure 2).

For TNF-α, the analysis incorporated 96 participants from three studies. The pooled result also disclosed no significant effect of sertraline, with an MD of −0.84 (95% CI: −4.04, 2.37; Z = 0.51, *p* = 0.61). Sizable heterogeneity was detected (I^2^ = 72%), indicating variability in the size and direction of study-specific effects (Figure 2).

Overall, the pooled results suggest that sertraline did not significantly alter circulating levels of IL-6, IL-10, or TNF-α across the included studies. Yet, the high heterogeneity across all three analyses indicates that these results should be interpreted with care, as study differences may have prejudiced the pooled estimates.

### 3.4. Risk of Bias

**Figure 3:**
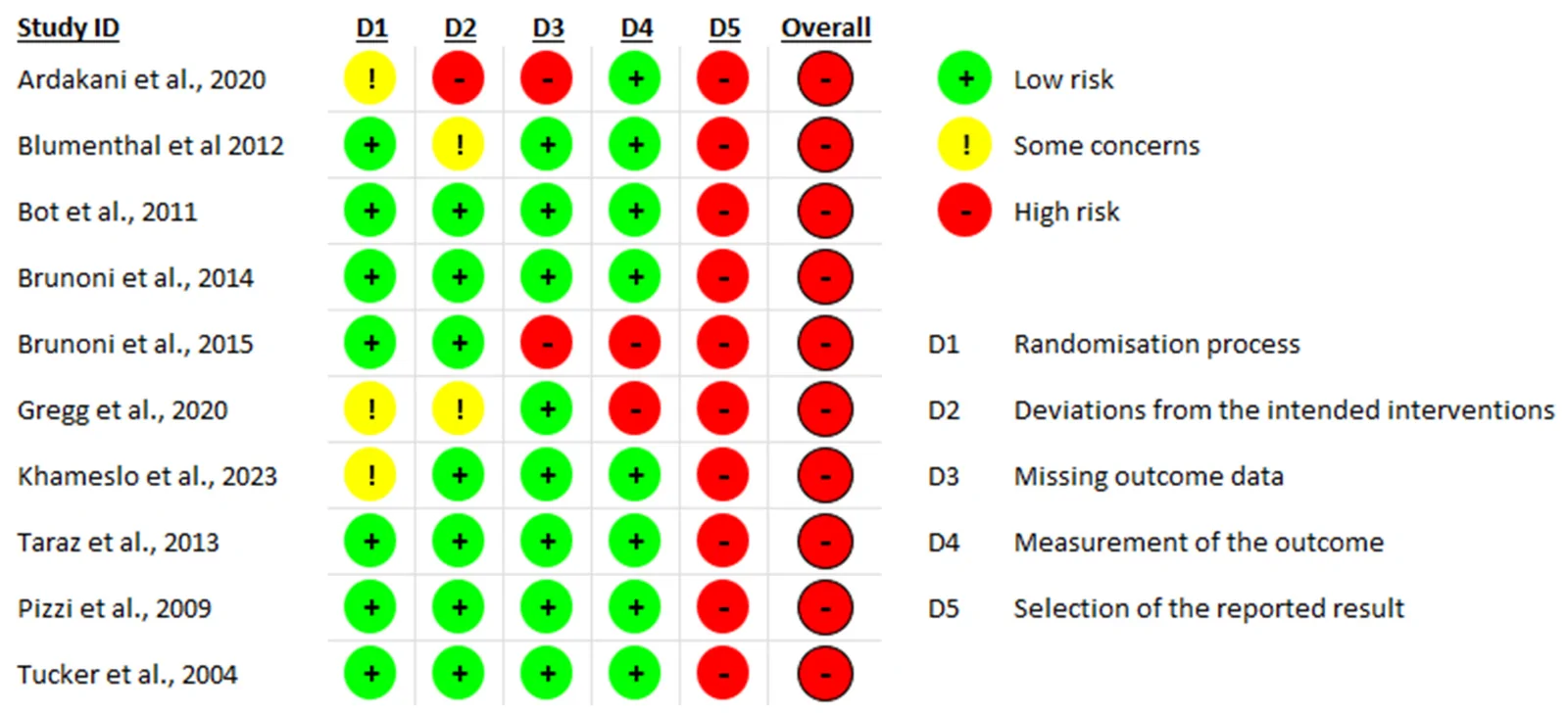
Risk-of-bias assessment of the included studies using RoB 2. Green, yellow, and red indicate low risk, some concerns, and high risk, respectively. The meaning and interpretation of domains D1–D5, together with the main reasons underlying the judgments, are described in Section 3.4.1, Section 3.4.2, Section 3.4.3, Section 3.4.4, Section 3.4.5 and Section 3.4.6 [23,24,25,26,34,35,36,37,38,39].

The risk of bias across the included studies demonstrated imperative methodological concerns, mainly in domains related to deviations from intended interventions and selection of reported results. Overall, all studies were judged as exhibiting a high risk of bias (Figure 3), chiefly by consistent issues in specific domains.

#### 3.4.1. Randomization Process (D1)

Most studies were judged as low risk for the randomization process, signifying that suitable random sequence generation and allocation procedures were normally implemented. But, some studies [24,25,37] presented some concerns, signifying incomplete reporting or potential limitations in allocation concealment.

#### 3.4.2. Deviations from Intended Interventions (D2)

The majority of studies were categorized as low risk in this domain, indicating that participants usually adhered to the assigned interventions and that proper analytical approaches were enforced. Still, some studies [25,34] highlighted some concerns, while Ardakani et al. [24] was categorized as high risk potentially owing to limited blinding or lack of reporting about adherence.

#### 3.4.3. Missing Outcome Data (D3)

Most studies demonstrated low risk for absent outcome data, suggesting that attrition was either minimal or correctly handled. Still, Brunoni et al. [36] and Ardakani et al. [24] were judged as high risk, indicating that absent data may have prejudiced the results or were not effectively addressed.

#### 3.4.4. Measurement of the Outcome (D4)

Several studies were rated as low risk for outcome measurement, reflecting the use of standardized and appropriate assessment methods. Yet, several studies [25,36] were categorized as high risk, likely as a result of lack of blinding of outcome assessors or possible measurement bias.

#### 3.4.5. Selection of the Reported Result (D5)

All included studies were judged as high risk in this domain, indicating consistent concerns about selective reporting. None of the studies visibly described pre-specified analysis plans, increasing the probability that reported outcomes may have been selectively presented.

#### 3.4.6. Overall Bias

Considering all domains, all studies were categorized as presenting an overall high risk of bias. This was mostly driven by the universal high risk in the choice of reported results domain, accompanied by additional concerns in outcome measurement and absent data in some studies. These results emphasize the requirement for careful interpretation of the pooled results.

### 3.5. GRADE Assessment

The GRADE assessment specified that the quality of evidence for the effects of sertraline on inflammatory biomarkers was very low across all evaluated outcomes, with major concerns linked to risk of bias, inconsistency, indirectness, and imprecision (Table 2). The specific GRADE evaluations for each outcome were as follows:

- IL-6: Very low certainty
- IL-10: Very low certainty
- TNF-α: Very low certainty

**Table 2:** Levels of evidence analysis via (GRADE Working Group, 2004) [39].

Outcome	No. of Studies	Risk of Bias	Inconsistency	Indirectness	Imprecision	Certainty of Evidence
**IL-6**	5	Very serious ^a^	Very serious ^b^	Very serious ^c^	Very serious ^d^	Very low
**IL-10**	3	Very serious ^a^	Serious ^e^	Serious ^f^	Very serious ^d^	Very low
**TNF-α**	3	Serious ^g^	Serious ^e^	Serious ^f^	Very serious ^d^	Very low

^a^: Deviations from intended interventions, missing outcome data and selection of the reported result; ^b^: I^2^ > 75%; ^c^: Difference in population profile and treatment; ^d^: Moderate presence and absence of effect based on 95%CI; ^e^: I^2^ between 50 and 75%; ^f^: Difference in population profile and treatment; ^g^: Selection of the reported result.

For IL-6, the certainty of evidence was demoted owing to very serious risk of bias, very serious inconsistency, very serious indirectness, and very serious imprecision.

For IL-10, the evidence was also rated as very low as a result of very serious risk of bias, serious inconsistency, serious indirectness, and very serious imprecision.

For TNF-α, the certainty of evidence was judged to be very low due to serious risk of bias, serious inconsistency, serious indirectness, and very serious imprecision.

Overall, these results indicate that confidence in the estimated effects of sertraline on IL-6, IL-10, and TNF-α is very restricted, and the true effects may be substantially different from the observed estimates. Detailed justifications for each GRADE judgment are offered in Table 2.

## 4. Discussion

### 4.1. Summary of Main Findings

The present systematic review and meta-analysis assessed the effects of sertraline on circulating inflammatory cytokines in adults across randomized double-blind placebo-controlled trials. Generally, the pooled analyses established that sertraline did not significantly alter the levels of crucial inflammatory biomarkers, including IL-6, IL-10, and TNF-α. Even if some individual studies reported drops in inflammatory markers after sertraline treatment, these results were not consistently replicated across the included trials.

Also, substantial heterogeneity was detected in all meta-analyses, suggesting considerable variability amongst studies regarding participant characteristics, clinical conditions, intervention protocols, and inflammatory assessment methods. In addition, the certainty of evidence was rated as very low for all evaluated outcomes because of imperative methodological limitations, including high risk of bias, inconsistency, indirectness, and imprecision.

### 4.2. Interpretation of Results

The absence of significant pooled effects may suggest that the anti-inflammatory properties of sertraline are limited or highly context-dependent. However, experimental evidence has anticipated that selective serotonin reuptake inhibitors may modulate immune and inflammatory pathways via serotonergic signaling, neuroendocrine regulation, and cytokine production [40,41]. Experimental models further demonstrate that environmental stressors can induce measurable cardiac structural changes that may be attenuated by pharmacological intervention [42].

A possible account is that inflammatory responses to sertraline may depend on factors such as baseline inflammatory status, disease severity, psychiatric diagnosis, and the presence of medical comorbidities. Additionally, changes in treatment duration and sertraline dosage may have prejudiced the variability in cytokine responses detected across studies. A further limitation is the difficulty of relating circulating cytokine concentrations to inflammatory activity within the brain. Peripheral and central cytokines originate from different cellular sources and are regulated within distinct biological compartments. Moreover, the blood–brain barrier, temporal differences in cytokine release, and local neuroimmune signaling limit the extent to which blood measurements reflect cytokine levels in the central nervous system. Thus, circulating cytokines should not be interpreted as direct indicators of brain inflammation.

Another important deliberation is that several included studies confirmed improvements in depressive symptoms without equivalent changes in inflammatory biomarkers. This result advocates that the therapeutic effects of sertraline may occur independently of systemic cytokine modulation. Alternatively, sertraline may exert localized neuroimmune effects within the central nervous system that are not sufficiently reflected by circulating peripheral biomarkers.

Controlled preclinical and experimental studies provide a useful framework for interpreting this discrepancy. In a chronic unpredictable mild stress model and in TNF-α-stimulated BV2 microglial cells, sertraline reduced inflammatory activation by decreasing TNF-α and IL-1β, and induced nitric oxide (iNOS) expression by inhibiting Nuclear Factor kappa B/κB-α inhibitor (NF-κB/IκB-α signaling), suggesting a direct effect on microglial inflammatory pathways [43]. Similarly, ex vivo experiments in human T lymphocytes showed that sertraline suppressed T-cell proliferative activity and reduced TNF-α secretion, with associated changes in Signal Transducer and Activator of Transcription 3 (STAT3) and Cyclooxygenase-2 (COX-2) expression, supporting a potential immunomodulatory action at the cellular level [18]. In rat hippocampus, sertraline also reduced basal IL-1β and TNF-α mRNA expression and attenuated the increase in these cytokines induced by seizures and lipopolysaccharide, which supports the possibility of localized central anti-inflammatory effects [20]. In addition, sertraline reversed oxidative damage and mitochondrial enzyme dysfunction in a 3-nitropropionic acid rat model, suggesting that some of its neuroprotective actions may involve antioxidant and mitochondrial mechanisms rather than direct systemic cytokine suppression alone [21].

These experimental findings support the biological plausibility of sertraline-related neuroimmune modulation, but they also help explain why the present clinical results were inconsistent. Preclinical models allow tighter control of inflammatory stimuli, dose, timing, tissue sampling, comorbidities, environmental exposures, and concomitant treatments. In contrast, the trials included in this review involved heterogeneous clinical populations, different treatment durations and sertraline dosages, variable baseline inflammatory states, and distinct medical comorbidities. Therefore, reductions in inflammatory mediators observed under controlled experimental conditions should be interpreted as mechanistic support for context-dependent effects rather than as direct evidence that sertraline reliably reduces circulating cytokines in clinical practice.

Moreover, inflammatory markers are affected by several physiological and lifestyle-related factors, including obesity, smoking, physical activity, sleep quality, and stress exposure [44], which were not consistently controlled across studies. So, the current evidence does not validate the use of sertraline as a dependable anti-inflammatory intervention in clinical practice.

### 4.3. Comparison with Previous Literature

The results of this review are somewhat consistent with previous research literature investigating the immunomodulatory effects of antidepressants, mostly selective serotonin reuptake inhibitors. Previous studies have suggested that selective serotonin reuptake inhibitors may exert modest anti-inflammatory effects, particularly by reductions in pro-inflammatory cytokines, for example, IL-6 and TNF-α [45,46]. Still, the available evidence has remained inconsistent, with some studies signifying significant biomarker reductions and others reporting no meaningful deviations despite clinical improvement in depressive symptoms.

In the present review, some individual trials stated reductions in inflammatory markers following sertraline treatment, chiefly in populations with cardiovascular disease, hemodialysis, or elevated baseline inflammation. Equally, other studies revealed no significant effects on cytokine levels or observed inflammatory changes that were analogous between sertraline and placebo groups [25,34]. These inconsistencies reinforce the complexity of the relationship between depression, inflammation, and antidepressant treatments.

Generally, the current results contribute to the growing body of evidence signifying that the anti-inflammatory effects of SSRIs are not uniform and should be interpreted carefully.

### 4.4. Heterogeneity, RoB 2 and GRADE

The heterogeneity observed across the pooled analyses was substantial to considerable for IL-6, IL-10, and TNF-α, as confirmed by the elevated I^2^ values for IL-6 (86%), IL-10 (72%), and TNF-α (72%). Several potential sources may explain this variability. First, chief clinical heterogeneity was identified among the included populations, which comprised patients with MDD, cardiovascular disease, CKD, hemodialysis, post-cardiovascular surgery conditions, post-traumatic stress disorder, and hematopoietic stem cell transplantation. Differences in baseline inflammatory status, comorbidities, and disease severity may likewise have prejudiced cytokine responses to sertraline treatment.

Intervention-related factors possibly contributed to the observed heterogeneity too. Considerable variability existed regarding sertraline dose, treatment duration, and therapeutic context. Whereas some studies evaluated sertraline as monotherapy, others combined sertraline with interventions such as aerobic exercise [47], which may have independently affected inflammatory outcomes.

Further heterogeneity arose from outcome-related methodological differences. The included studies assessed distinct cytokines and inflammatory biomarkers using different laboratory methods, timing of blood collection, and approaches for reporting post-intervention values. Also, methodological heterogeneity was evident in sample size, follow-up duration, statistical analysis strategies, and management of absent data. The relatively small number of studies available for each outcome also restricted the feasibility of subgroup and sensitivity analyses.

Such population heterogeneity reduces the reliability, precision, and clinical interpretability of the pooled estimates. Combining participants with markedly different disorders, comorbidity profiles, baseline inflammatory activity, and disease severity assumes that sertraline has a sufficiently comparable biological effect across clinical settings. This assumption may not be valid because cytokine concentrations and treatment responsiveness are strongly influenced by the underlying condition, concomitant medications, organ dysfunction, and the intensity of systemic inflammation. As a result, the pooled estimates may average genuinely different, or even opposing, treatment effects and may not accurately represent any single patient population. Although the random-effects model accounts statistically for between-study variability, it cannot remove the clinical differences responsible for that variability. Moreover, the limited number of studies prevented robust subgroup or meta-regression analyses to determine whether diagnosis, baseline inflammation, or other population characteristics modified the effects of sertraline. Consequently, the nonsignificant pooled findings should not be interpreted as definitive evidence that sertraline lacks anti-inflammatory activity. Instead, they indicate that the available evidence is inconsistent and insufficiently homogeneous to support a reliable estimate of its effects across specific clinical populations.

This extensive clinical and methodological heterogeneity represents the principal limitation of the evidence base. Assessed differences in underlying diagnoses, comorbidity profiles, treatment duration, concomitant interventions, and cytokines substantially reduce direct comparability across studies. Consequently, the pooled estimates may reflect heterogeneous biological and clinical contexts rather than a consistent effect of sertraline. These limitations weaken the precision, interpretability, and generalizability of the meta-analytic findings.

The RoB 2 assessment established that all included studies presented an overall high risk of bias. This classification was primarily driven by concerns regarding selective reporting of results, while additional issues related to absent outcome data and outcome measurement were also identified in some studies. Thus, these methodological limitations reduce confidence in the pooled estimates, and the absence of statistically significant effects should not be interpreted as definitive evidence that sertraline has no impact on inflammatory biomarkers.

Consistently, the GRADE assessment indicated a very low certainty of evidence for IL-6, IL-10, and TNF-α outcomes, mainly because of serious concerns about risk of bias, inconsistency, indirectness, and imprecision. From a clinical perspective, the current evidence remains insufficient to conclude that sertraline reliably modulates systemic inflammation. So, any likely anti-inflammatory effects of sertraline may be context-dependent and influenced by patient characteristics, underlying clinical conditions, and methodological factors.

### 4.5. Clinical Implications

We underline that sertraline should remain principally a psychiatric treatment. Even though sertraline is clinically effective for improving depressive symptoms, the current evidence does not support its use as a dependable anti-inflammatory intervention. In the present systematic review and meta-analysis, the pooled results did not establish significant effects on key inflammatory cytokines, including IL-6, IL-10, and TNF-α.

Another important consideration is that inflammatory status alone should not guide sertraline prescription in clinical practice. Clinicians should not recommend sertraline with the expectation of consistently reducing systemic inflammation. Though some individual studies reported reductions in inflammatory biomarkers, these results appear to be context-dependent and may be prejudiced by baseline inflammatory status, comorbidities, disease severity, treatment duration, and concomitant interventions.

We similarly emphasize that biomarker monitoring currently appears to be more relevant in the research setting than in routine clinical practice. While cytokine assessment may contribute to upcoming mechanistic or stratified clinical trials, the available evidence does not justify the routine measurement of inflammatory biomarkers in patients receiving sertraline treatment.

From a practical perspective, clinical decision-making should continue to prioritize established therapeutic outcomes, including improvement in depressive symptoms, treatment tolerability, safety profile, and patient-specific psychiatric needs.

### 4.6. Strengths and Limitations of the Study

One of the main assets of this review is its focused research question, explicitly evaluating the effects of sertraline on inflammatory cytokines. In addition, only randomized placebo-controlled trials were included, which reinforces the internal validity of the results compared with observational evidence.

An important strength of this review lies in its comprehensive and systematic search strategy, which spanned multiple databases: Lilacs, CINAHL, MEDLINE/PubMed, Cochrane, Scopus, and Web of Science. Also, the methodology upholds the highest standards of transparency and reproducibility, as the review adheres to PRISMA recommendations and was registered in the PROSPERO database.

The independent study selection and data extraction process also supported the methodological rigor of the review, as no less than two reviewers independently screened studies and extracted data, with disagreements resolved by additional reviewers when needed.

However, several restrictions should also be recognized. The relatively small number of studies available for each outcome limited the feasibility of subgroup analyses, sensitivity analyses, and a more robust exploration of publication bias. Additionally, differences in cytokine measurement techniques, timing of blood collection, and reporting formats across studies may have reduced comparability among trials and contributed to the observed heterogeneity.

### 4.7. Future Directions

Forthcoming studies should prioritize well-designed, adequately powered randomized placebo-controlled trials to elucidate whether sertraline has clinically meaningful immunomodulatory effects. These trials should use rigorous randomization techniques, allocation concealment, blinding, pre-specified protocols, and transparent statistical analysis plans to reduce the risk of bias and selective outcome reporting.

Future research should also standardize the assessment and reporting of inflammatory outcomes, including the type of cytokines measured, laboratory methods, timing of blood collection, units of measurement, and reporting of baseline and post-intervention values. This would advance comparability across studies and facilitate a stronger meta-analysis.

One more important route is the stratification of participants consistent with baseline inflammatory status, disease severity, comorbidities, and clinical diagnosis. Since the included studies involved heterogeneous populations, future trials should study whether specific subgroups, for example patients with elevated baseline inflammation, cardiovascular disease, CKD, or treatment-resistant depression, may be more likely to experience inflammatory changes after sertraline treatment.

Longer follow-up periods are also required to determine whether the potential effects of sertraline on inflammatory biomarkers are transient, delayed, or dependent on treatment duration. Also, future studies should appraise whether changes in cytokines are linked with improvements in depressive symptoms, functional outcomes, cardiovascular markers, or other clinically relevant endpoints.

Finally, future research should extricate the isolated effects of sertraline from those of concomitant interventions, for instance exercise or transcranial direct current stimulation. Trials designed to compare sertraline monotherapy with placebo, besides factorial studies with adequate power, may help determine whether any observed anti-inflammatory effects are directly attributable to sertraline or reflect broader clinical improvement, placebo effects, or combined therapeutic strategies.

## 5. Conclusions

In summary, the available evidence does not demonstrate a significant or consistent effect of sertraline on circulating IL-6, IL-10, or TNF-α. Although reductions were reported in some individual studies, the pooled estimates were not statistically significant and were accompanied by substantial heterogeneity. This heterogeneity reflects major differences in clinical populations, underlying diseases, treatment duration, concomitant interventions, and cytokines assessed, which limits the comparability and generalizability of the findings. Confidence in the estimates is further reduced by the high risk of bias, inconsistency, indirectness, and imprecision, resulting in very low certainty of evidence. Sertraline should consequently be viewed primarily as a psychiatric treatment, while any anti-inflammatory effect remains uncertain. More rigorous randomized trials using standardized populations, treatment protocols, cytokine measurements, and longer follow-up are needed.

## Figures and Tables

**Table 1:** Description of the characteristics of the study population of articles by author and year, sample and main results.

Author/ Years	Sample	Main Results
Ardakani et al. 2020 [24]	Sertraline group: n = 24 Placebo group: n = 20	IL-6: Significant reduction in serum IL-6 levels after 8 weeks in the sertraline group compared with baseline and placebo Changes from baseline to week 8 were significantly greater in the sertraline group (*p* = 0.048). IL-10: No significant inter-group or intra-group differences throughout the study period.
Blumenthal et al. 2012 [34]	Sertraline group: n = 40 Placebo group: n = 24	IL-6: No significant reduction compared with placebo. Overall, sertraline did not produce significant improvements in inflammatory cytokine markers compared with placebo.
Bot et al. 2011 [26]	Total sample size: n = 122	IL-6, and TNF-α did not predict response or remission following sertraline treatment. No significant differences in baseline cytokine levels between responders and non-responders. Changes in cytokine levels over 10 weeks were not consistently associated with changes in depressive symptoms.
Brunoni et al. 2014 [35]	Sertraline group: n = 18 Placebo group: n = 19	Cytokines assessed: IL-2, IL-4, IL-6, IL-10, IL-17A, IFN-γ, TNF-α Findings: Plasma levels of all cytokines except TNF-α decreased over time during treatment No significant sertraline-specific effect compared with placebo.
Brunoni et al. 2015 [36]	Sertraline group: n = 18 Placebo group: n = 19	Biomarkers assessed: Soluble TNF receptors 1 and 2 (sTNFR1, sTNFR2) Findings: Plasma levels of sTNFR1 and sTNFR2 did not change over time No significant differences between sertraline and placebo Baseline sTNFR levels did not predict antidepressant response.
Gregg et al. 2020 [25]	Sertraline group: n = 97 Placebo group: n = 96	Inflammatory biomarkers assessed: High-sensitivity IL-6, Albumin, Pre-albumin Key findings: IL-6 did not predict response to sertraline Sertraline did not significantly reduce inflammatory biomarker levels over time; biomarkers functioned as predictors of response, not treatment-mediated changes.
Khameslo et al. 2023 [37]	Placebo: n = 8 Sertraline: n = 8	Sertraline alone significantly reduced IL-6 and TNF-α compared with placebo.
Pizzi et al. 2009 [38]	Sertraline: n = 47 Placebo: n = 48	Sertraline treatment led to a significant reduction in IL-6 levels after 20 weeks compared with both baseline and placebo.
Taraz et al. 2013 [23]	Sertraline: n = 25 Placebo: n = 25	IL-6: Significantly decreased in the sertraline group compared with placebo at week 12 TNF-α: Decreased within the sertraline group, but no significant between-group difference at week 12 IL-10: Increased within the sertraline group; no significant difference versus placebo.
Tucker et al. 2004 [39]	Sertraline group: n = 18 Placebo: n = 7	IL-1β: Significantly decreased from baseline after sertraline treatment IL-2R: Significantly increased after sertraline treatment.

## Data Availability

No new data were created or analyzed in this study. Data sharing is not applicable to this article.

## Supplementary Materials

The following supporting information can be downloaded at: https://www.mdpi.com/article/10.3390/medsci14040460/s1, Table S1: PRISMA 2020 Checklist; Table S2: Search strategy for details.
