# Supplementary material for: Sertraline as an Antidepressant and Inflammatory Cytokines in Depressive Disorders: A Systematic Review and Meta-Analysis of Randomized Double-Blind Placebo-Controlled Trials
Source: Med Sci (Basel). 2026 Aug 6;14(4):460. doi: 10.3390/medsci14040460 (PMC13515890; doi:10.3390/medsci14040460)
Supplement: Supplementary file 1 [file medsci-14-00460-s001.zip › medsci-4414394-Table S2.pdf]

Database	Search Strategy
PUBMED	(("Sertraline"[MeSH Terms] OR Sertraline OR Zoloft OR Lustral OR Altruline OR Gladem OR Sealdin)<br><br>AND<br><br>("Cytokines"[MeSH Terms] OR cytokine* OR interleukin* OR "inflammatory marker*" OR "tumor necrosis factor" OR TNF OR IL-6 OR IL-1β OR CRP)
LILACS	(Sertraline OR Zoloft OR Lustral OR Altruline OR Gladem OR Sealdin)<br><br>AND<br><br>(cytokine OR interleukin OR "inflammatory marker" OR IL-6 OR IL-1β OR CRP OR TNF)
Cochrane Library	(Sertraline OR Zoloft OR Lustral OR Altruline OR Gladem OR Sealdin)<br><br>AND<br><br>(cytokine OR interleukin OR "inflammatory marker" OR IL-6 OR IL-1β OR CRP OR TNF)
SCOPUS	TITLE-ABS-KEY(Sertraline OR Zoloft OR Lustral OR Altruline OR Gladem OR Sealdin)<br><br>AND<br><br>TITLE-ABS-KEY(cytokine OR interleukin OR "inflammatory marker" OR IL-6 OR IL-1β OR TNF OR CRP)<br><br>AND<br><br>TITLE-ABS-KEY(depression OR "depressive disorder")
Web of Science (Coleção principal)	TS=(Sertraline OR Zoloft OR Lustral OR Altruline OR Gladem OR Sealdin)<br><br>AND<br><br>TS=(cytokine OR interleukin OR "inflammatory marker" OR IL-6 OR IL-1β OR TNF OR CRP)<br><br>AND<br><br>TS=(depression OR "depressive disorder")

CINAHL	((MH "Sertraline" OR Sertraline OR Zoloft OR Lustral OR Altruline OR Gladem OR Sealdin)<br><br>AND<br><br>(MH "Cytokines" OR cytokine* OR interleukin* OR "inflammatory marker*" OR "tumor necrosis factor" OR TNF OR IL-6 OR IL-1β OR CRP)
